# Immune exclusion as a recurrent immune-escape state driving treatment resistance in osteosarcoma: insights from single-cell, spatial, and multi-omics studies

**DOI:** 10.3389/fimmu.2026.1802194

**Published:** 2026-03-24

**Authors:** Yunxia Zeng, Yiyao Xiang, Lang Chen, Yuan Wen, Changjie Deng, Xinyu Huang, Lei Feng, Shili Liu, Heng Zhao

**Affiliations:** 1 Department of Orthopaedics, The Fourth Affiliated Hospital of Southwest Medical University, Meishan, China; 2 Department of Orthopaedics, The Affiliated Hospital of Southwest Medical University, Luzhou, China; 3 Department of Emergency Medicine, The Affiliated Hospital of Southwest Medical University, Luzhou, China; 4 Health Management Center, The Fourth Affiliated Hospital of Southwest Medical University, Meishan, China; 5 Department of Medical Equipment, The Fourth Affiliated Hospital of Southwest Medical University, Meishan, China; 6 Intensive Care Unit, The Affiliated Hospital of Southwest Medical University, Luzhou, China; 7 Intensive Care Unit, The Fourth Affiliated Hospital of Southwest Medical University, Meishan, China

**Keywords:** immune exclusion, multi-omics integration, osteosarcoma, single-cell sequencing, treatment resistance, tumor immune microenvironment

## Abstract

Osteosarcoma remains a therapeutically challenging malignancy with durable responses limited by frequent treatment resistance. Although immune activity is detectable in many tumors, immunotherapy and combination strategies show limited clinical benefit, indicating immune dysfunction extends beyond tumor-intrinsic mechanisms. Growing evidence identifies immune exclusion as a key immunological barrier in osteosarcoma, characterized by spatial and functional segregation of immune cells from malignant areas despite their presence in the tumor microenvironment. Here, we use immune exclusion as a working term to describe contexts in which immune cells are present but have limited effective access to, or engagement with, malignant cells—distinct from immune desert (near-absence of infiltrates) and immune-cold states (weak effector activation despite some infiltration). In this mini-review, we focus on immune exclusion as a context-dependent immunological driver of treatment resistance in osteosarcoma. We integrate insights from single-cell sequencing, spatial profiling, and multi-omics studies to characterize the immunological features of immune exclusion, highlight bone-associated structural and regulatory factors, and explore how exclusion creates selective pressure leading to therapeutic failure. Emerging data indicate that immune exclusion represents a coordinated program involving tumor, stromal, and immune elements, resulting in impaired immune–tumor interaction and ineffective clearance. We discuss the translational implications, stressing immune-context modulation as essential for re-sensitization and noting that many current single-cell and spatial datasets remain limited in sample size, sampling sites, and treatment background. Framing resistance through immune exclusion offers an immune-centric framework for understanding and overcoming treatment resistance in osteosarcoma.

## 1 Introduction

Osteosarcoma remains a therapeutically challenging malignancy in which durable responses are difficult to achieve despite aggressive multimodal treatment (1). Although chemotherapy can control localized disease in some patients, relapse and metastatic progression are frequent, and immunotherapy has shown limited and inconsistent benefit compared with its success in other solid tumors (2–4). These observations suggest that treatment resistance in osteosarcoma cannot be fully explained by tumor-intrinsic mechanisms alone and instead reflects fundamental constraints imposed by the tumor immune microenvironment.

Accumulating evidence indicates that immune exclusion represents a prominent and recurrent (rather than uniformly “dominant”) immunological barrier in osteosarcoma (5). Immune exclusion describes a state in which immune cells are present but spatially or functionally segregated from malignant cells, preventing effective immune-mediated cytotoxicity (6). This phenotype differs from immune-cold or immune-ignorant tumors in that excluded lesions often show immune infiltration confined to stromal or peripheral compartments. In osteosarcoma, immune exclusion is especially pertinent because the bone-associated microenvironment—marked by a dense extracellular matrix, aberrant vasculature, and stromal programs—can collectively restrict immune cell access to tumor cores (7).

From an immunological perspective, immune exclusion has direct implications for treatment resistance (8). When immune surveillance is spatially restricted, cytotoxic therapies may shrink tumor burden yet fail to facilitate immune-mediated clearance, thereby permitting the persistence and expansion of resistant tumor states (9). Recent advances in single-cell and multi-omics profiling now allow immune exclusion to be examined at cellular and regulatory resolution, revealing coordinated tumor, stromal, and immune programs that sustain immune inaccessibility (10).

In this mini-review, we focus on immune exclusion as a specific immunological driver of treatment resistance in osteosarcoma. By integrating insights from single-cell and multi-omics studies, we aim to clarify how exclusionary immune states emerge, how they condition therapeutic failure, and how targeting immune accessibility may inform re-sensitization strategies.

## 2 Immune exclusion in osteosarcoma: immunological definition and disease-specific context

### 2.1 Immune exclusion as a distinct immunological state

In tumor immunology, immune exclusion defines a state in which immune cells are present within the tumor microenvironment but are spatially or functionally prevented from engaging malignant cells (8). This phenotype is fundamentally distinct from immune-cold or immune-desert tumors, where immune infiltration is globally absent (6). In immune-excluded tumors, immune components—most notably T cells and myeloid cells—are often detectable at the tumor periphery, within stromal regions, or along vascular structures, yet fail to penetrate tumor cell–dense compartments (11).

**Figure 1:**
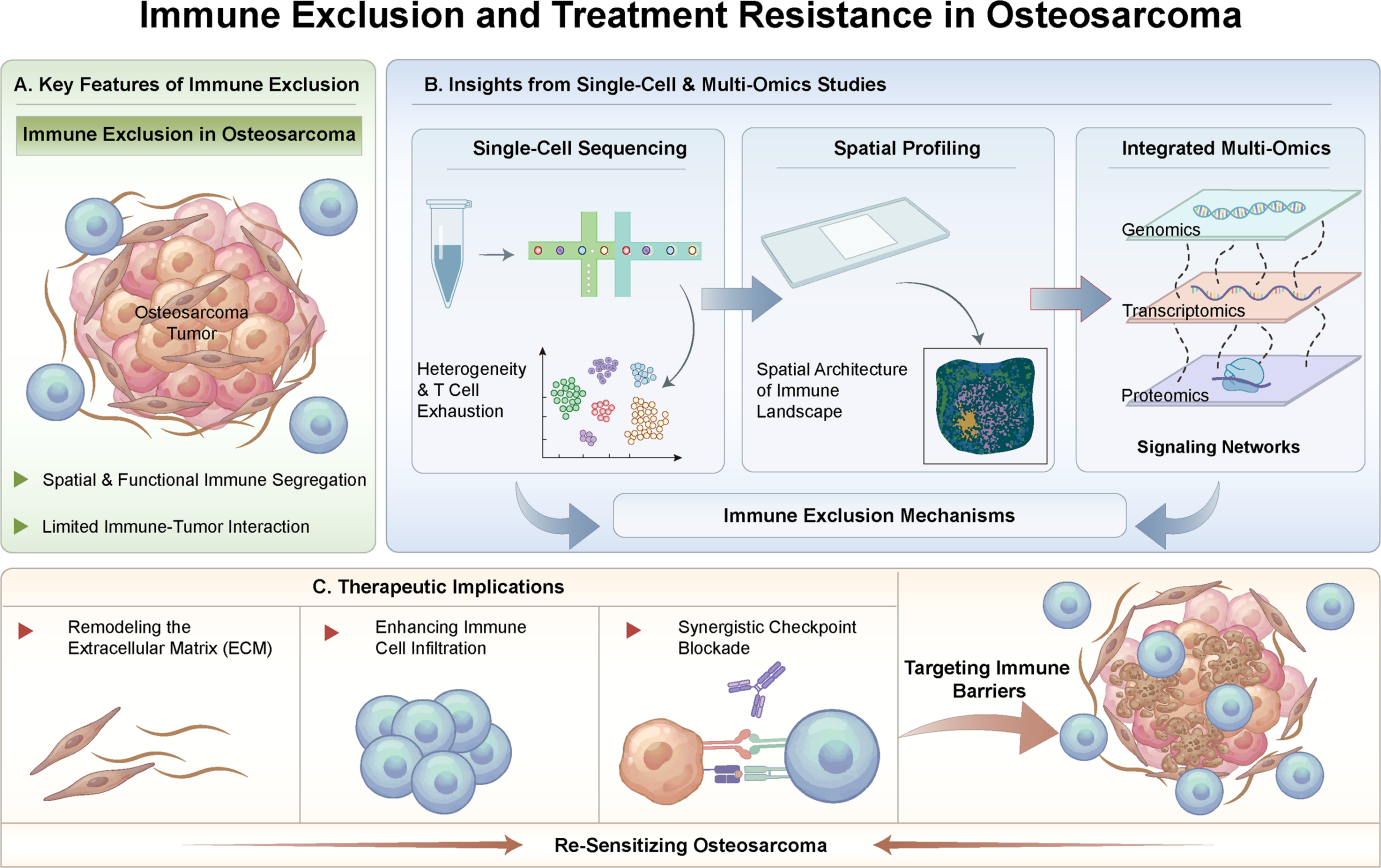
Immune exclusion and treatment resistance in osteosarcoma. **(A)** Overview of immune exclusion as a spatial barrier that limits immune–tumor contact and promotes resistance. **(B)** Single-cell, spatial, and multi-omics approaches delineate immune and stromal heterogeneity, map compartmentalized immune localization, and nominate exclusion-associated programs. **(C)** Therapeutic implications include remodeling immune barriers, enhancing infiltration, and rational combination strategies to re-sensitize osteosarcoma.

This distinction is particularly important in osteosarcoma, where immune activity is frequently detectable but rarely translates into effective tumor control (12). Bulk immune signatures can indicate immune cell presence, yet clinical responses to immunotherapy remain limited. Immune exclusion offers a mechanistic rationale for this mismatch by shifting the emphasis from immune abundance to immune accessibility. Accordingly, the spatial organization of immune cells—rather than their overall quantity—emerges as the key determinant of immune effectiveness (**Figure 1**).

### 2.2 Bone-associated microenvironment as a structural basis for immune exclusion

Osteosarcoma arises within a highly specialized tissue environment that imposes unique physical and biological constraints on immune cell behavior (4). The osteoid-rich extracellular matrix, characteristic of malignant osteoblastic tumors, creates a dense and rigid stromal architecture that limits immune cell migration (13). In addition, aberrant vasculature and altered interstitial pressure within bone tumors further restrict immune trafficking and retention within tumor cores (14).

Unlike many epithelial tumors, where immune exclusion is often driven by desmoplasia or fibroblast-dominated stroma, immune exclusion in osteosarcoma is reinforced by bone-specific structural elements (15). These features create a microenvironment in which immune cells can be recruited but remain spatially segregated, favoring peripheral accumulation rather than intratumoral infiltration. As a result, immune exclusion in osteosarcoma is not an incidental phenomenon but a predictable consequence of tissue context.

### 2.3 Active immunological programs sustaining exclusionary architecture

Immune exclusion in osteosarcoma cannot be attributed solely to physical barriers. Single-cell studies indicate that exclusionary states are actively maintained by coordinated immunological programs involving tumor cells, stromal compartments, and immune infiltrates (16). Tumor cells may downregulate antigen presentation pathways or attenuate interferon responsiveness, reducing immune recognition within tumor cores (17). Concurrently, stromal and myeloid populations express chemokine and cytokine profiles that bias immune cell localization toward non-tumor regions (18).

These programs generate a microenvironment in which immune cells are not simply blocked but redirected or functionally constrained. Cytotoxic T cells may persist in dysfunctional or non-cytolytic states, while suppressive myeloid populations reinforce immune mislocalization and dampen effector function (19). Together, these processes stabilize immune exclusion as a state-level property of the tumor microenvironment rather than a transient or passive condition.

### 2.4 Immunological consequences of immune exclusion in osteosarcoma

The immunological consequence of immune exclusion is not immune silence, but immune inefficacy. Immune cells remain present and transcriptionally active, yet limited access to tumor cells prevents effective immune surveillance and clearance. This setting creates a permissive niche in which tumor cells can persist under immune pressure without requiring complete immune evasion.

In osteosarcoma, this exclusionary immune architecture provides a foundation for subsequent treatment resistance (20). By restricting immune–tumor interactions, immune exclusion limits the immune contribution to therapy-induced tumor elimination, thereby setting the stage for incomplete responses and residual disease persistence. Thus, immune exclusion represents a foundational immunological state that precedes and enables resistance, rather than a downstream consequence of therapeutic failure.

## 3 Single-cell and spatial evidence for immune exclusion in osteosarcoma

### 3.1 Single-cell resolution of immune dysfunction in excluded tumors

Bulk immune profiling has long suggested that osteosarcoma is not immunologically inert; however, such analyses obscure the cellular states that determine immune effectiveness (21). Single-cell transcriptomic studies have clarified this discrepancy by revealing that immune cells within osteosarcoma frequently occupy functionally constrained states consistent with immune exclusion (22). Cytotoxic T lymphocytes are commonly detected but exhibit transcriptional features associated with impaired effector differentiation, attenuated interferon responsiveness, or early exhaustion, indicating that immune presence does not equate to immune competence (21).

Importantly, these dysfunctional immune states are not uniformly distributed across tumors (23). Single-cell data reveal that immune impairment often coexists with preserved immune signaling in adjacent stromal or perivascular compartments, suggesting that immune dysfunction is shaped by microenvironmental context rather than intrinsic immune cell deficiency (24). This observation supports immune exclusion as a spatially conditioned immunological state in which immune cells are present but rendered ineffective by their localization and surrounding signals.

### 3.2 Spatial compartmentalization as a defining feature of immune exclusion

Spatially informed analyses offer the most direct evidence for immune exclusion in osteosarcoma. Across multiple studies, immune cells—including T cells and antigen-presenting cells—are preferentially distributed within stromal regions, perivascular niches, or tumor margins, with only limited infiltration into tumor cell–dense, osteoid-rich cores (25). This compartmentalization is observed even in tumors with relatively high immune cell abundance, highlighting that immune exclusion is defined by immune positioning rather than immune quantity.

In osteosarcoma, spatial segregation is further reinforced by bone-specific features, including dense extracellular matrix deposition and irregular vascular organization (26). These structural characteristics partition the lesion into immune-accessible and immune-inaccessible zones. As a result, immune cells can remain transcriptionally active and phenotypically identifiable while being physically separated from malignant cells, thereby limiting effective immune–tumor engagement. Spatial analyses thus shift immune exclusion from an inferred construct to an observable architectural property of the tumor microenvironment.

### 3.3 Coordinated tumor–stroma–immune programs underlying exclusion

Single-cell and spatial datasets further indicate that immune exclusion in osteosarcoma is sustained by coordinated cross-compartmental programs rather than isolated cellular defects. Tumor cells within excluded regions frequently display transcriptional signatures consistent with reduced antigen presentation and dampened interferon signaling, diminishing immune recognition within tumor cores (27). Stromal populations, including fibroblast-like and endothelial cells, express chemokine and extracellular matrix programs that bias immune cell localization toward non-tumor compartments (28).

These coordinated programs create a microenvironmental logic in which immune cells are actively retained or redirected away from malignant regions. Immune exclusion thus reflects an organized immunological architecture shaped by reciprocal signaling between tumor, stromal, and immune compartments, rather than a passive failure of immune recruitment.

### 3.4 Heterogeneity and plasticity of immune exclusion states

An important insight from single-cell analyses is that immune exclusion in osteosarcoma is heterogeneous and potentially plastic. Different lesions—and even distinct regions within the same tumor—can exhibit varying degrees of immune accessibility. Some tumors display partial immune penetration accompanied by rapid immune dysfunction, whereas others show near-complete spatial segregation with minimal immune–tumor interface (29).

This heterogeneity has important implications for treatment response and therapeutic design. Immune exclusion should be considered a spectrum of states rather than a strictly binary condition. Determining where individual tumors fall along this spectrum is crucial for anticipating therapeutic vulnerability and for tailoring strategies intended to restore immune access. Single-cell and spatial profiling offer the resolution needed to delineate this continuum and to pinpoint transition points that may be amenable to intervention.

## 4 Immune exclusion as an immunological selective pressure driving treatment resistance

### 4.1 Immune exclusion disrupts immune–therapy coupling

Therapeutic efficacy in cancer is increasingly understood as the product of direct tumor cell killing and immune-mediated reinforcement (30). Cytotoxic chemotherapy can promote immunogenic cell death, while immune-based therapies rely on sustained immune–tumor contact to amplify effector responses (31). In osteosarcoma, immune exclusion disrupts this coupling by spatially separating immune cells from malignant compartments, thereby attenuating immune participation in therapeutic response (32).

When immune cells are excluded from tumor cores, therapy-induced tumor debulking occurs in the absence of effective immune surveillance (33). This decoupling converts treatment into an incomplete selective pressure: sensitive tumor populations may be eliminated, yet residual cells that survive initial stress remain shielded from immune-mediated clearance (34, 35). As a result, immune exclusion transforms therapy from a potentially curative intervention into a transient cytoreductive event, predisposing tumors to relapse and resistance.

### 4.2 Selection of immune-insensitive tumor states

Immune exclusion alters the evolutionary constraints acting on tumor cells (36). In immune-accessible environments, tumor cells that fail to present antigens or respond to interferon signaling are selectively eliminated (37). By contrast, in immune-excluded osteosarcoma, such immune-insensitive phenotypes can persist with reduced fitness costs (38). Single-cell and multi-omics analyses indicate that tumor cells within excluded regions are enriched for transcriptional states characterized by reduced antigen presentation, attenuated interferon responsiveness, and altered stress-response pathways (39, 40).

Importantly, these states do not necessarily represent fixed genetic resistance mechanisms (41). Instead, they reflect adaptive phenotypes that are tolerated—and in some cases potentially favored—within exclusionary immune contexts (42). Immune exclusion therefore can bias clonal selection toward tumor states that are compatible with immune invisibility or indifference, thereby accelerating the emergence of resistant disease without requiring overt immune escape mutations (43, 44).

### 4.3 Co-evolution of immune dysfunction and tumor resistance

Immune exclusion also shapes the evolution of immune compartments themselves, reinforcing resistance through reciprocal adaptation (45). Persistent spatial segregation and ineffective tumor engagement drive immune dysfunction, including exhaustion of cytotoxic lymphocytes and dominance of suppressive myeloid programs (46). These immune adaptations further weaken selective pressure against residual tumor cells, creating a self-reinforcing loop in which immune dysfunction and tumor resistance may co-evolve.

In osteosarcoma, this co-evolutionary process is particularly pronounced due to the stability of exclusionary architecture imposed by the bone-associated microenvironment. Once established, immune exclusion limits the capacity of both immune cells and therapies to reassert control, even in the presence of immune activation signals (47). Resistance thus emerges not as a late-stage aberration, but as an emergent property of an immune-restricted ecosystem (48).

### 4.4 Implications for interpreting resistance in osteosarcoma

Viewing immune exclusion as an immunological selective pressure reframes how resistance should be interpreted in osteosarcoma. Rather than attributing treatment failure solely to tumor-intrinsic adaptations, this framework emphasizes the role of immune accessibility in determining whether therapeutic pressure is effectively translated into durable tumor control (9, 49). Resistance, in this context, reflects the survival and adaptation of tumor states that evolve under conditions of limited immune engagement.

This perspective has direct implications for both biomarker development and therapeutic design (50). Molecular features associated with resistance should be interpreted in light of immune context, as identical tumor-intrinsic alterations may have divergent consequences depending on immune accessibility (51). By positioning immune exclusion upstream of resistance, this framework provides a unifying explanation for the limited success of intensified or combination therapies that fail to address immune architecture.

## 5 Multi-omics signatures of immune exclusion and translational implications

### 5.1 Transcriptional programs associated with immune inaccessibility

Transcriptomic analyses consistently suggest that immune exclusion in osteosarcoma is accompanied by coordinated suppression of immune-interactive programs rather than isolated gene-level alterations (52). Tumor cells within immune-excluded regions frequently show reduced expression of antigen processing and presentation machinery, attenuated interferon-stimulated gene responses, and altered chemokine signaling profiles that limit immune cell recruitment and retention (53). These transcriptional features collectively define an immune-inaccessible tumor state in which immune recognition is dampened despite the presence of immune cells in adjacent compartments.

Importantly, such programs are often reversible and context-dependent, suggesting that immune exclusion is maintained through regulatory adaptation rather than fixed genetic lesions (54). This distinction has practical implications: transcriptional signatures of exclusion may serve as dynamic indicators of immune accessibility and therapeutic vulnerability, rather than static predictors tied to mutational status.

### 5.2 Epigenetic stabilization of exclusionary immune states

Epigenetic regulation represents a key mechanism by which immune exclusion is stabilized in osteosarcoma (54). Chromatin accessibility and DNA methylation patterns linked to immune gene repression can constrain antigen presentation and interferon responsiveness, thereby reinforcing immune invisibility within tumor cores (43, 55). These epigenetic features provide a mechanistic explanation for the persistence of immune exclusion even in the presence of inflammatory or immune-activating signals.

From an immunological perspective, epigenetic stabilization of exclusionary states is particularly relevant to treatment resistance (56). Epigenetically constrained tumors may fail to re-engage immune surveillance following therapy-induced stress, thereby limiting immune-mediated clearance of residual disease (54, 57). As a result, epigenetic features associated with immune exclusion may represent both biomarkers of resistance and rational targets for immune-context reprogramming.

### 5.3 Metabolic constraints limiting immune cell fitness

Metabolic reprogramming adds an additional layer to immune exclusion that is increasingly captured by integrated multi-omics approaches (58). Tumor and stromal metabolic programs can generate nutrient-depleted, hypoxic, or acidic microenvironments that selectively impair immune cell survival and effector function (59, 60). In osteosarcoma, such metabolic constraints intersect with immune exclusion by reducing immune cell fitness within tumor cores, even when immune cells are transcriptionally primed for activation.

These metabolic features may not simply coexist with immune exclusion but can reinforce it by creating conditions that favor immune dysfunction and spatial mislocalization (61, 62). Accordingly, metabolic signatures linked to immune exclusion help explain why immune cells fail to persist or function effectively near malignant cells, further consolidating exclusionary architecture.

### 5.4 Integrated multi-omics signatures for stratification and therapeutic planning

The convergence of transcriptional, epigenetic, and metabolic programs defines immune exclusion as a composite molecular state rather than a single-pathway abnormality (63). Integrated multi-omics signatures that capture this state offer a practical framework for stratifying osteosarcoma according to immune accessibility and resistance risk (8, 64). Such signatures may outperform single-modality biomarkers by reflecting the coordinated nature of immune exclusion across tumor-intrinsic and microenvironmental dimensions.

From a translational standpoint, immune exclusion signatures have dual utility. First, they can identify patients unlikely to benefit from immune-based therapies without prior microenvironmental modulation (65). Second, they can guide therapeutic sequencing by indicating when immune priming or microenvironmental remodeling may be needed to enable effective immune engagement (66). In this context, multi-omics signatures should be interpreted not as endpoints, but as decision-support tools that guide immune-informed treatment design.

## 6 Discussion

This mini-review positions immune exclusion as a central immunological condition that shapes treatment resistance in osteosarcoma. Rather than indicating an absence of immune activity, immune exclusion describes a setting in which immune surveillance is detectable but spatially ineffective, thereby constraining productive immune–tumor interactions (67, 68). This framework helps explain the persistent discrepancy between detectable immune signals and the limited efficacy of immunotherapy and combination treatments in osteosarcoma.

A key conceptual distinction emerging from recent studies is that immune presence does not equate to immune accessibility. In immune-excluded osteosarcoma, immune cells may be transcriptionally active yet remain unable to engage malignant cells due to exclusionary architecture (69, 70). This spatially conditioned immune dysfunction reframes resistance as an immunological permissive state, in which therapeutic pressure is insufficiently coupled to immune-mediated tumor clearance (71). Consequently, resistance should be interpreted not solely as a tumor-intrinsic adaptation, but as a context-dependent outcome shaped by immune accessibility.

Single-cell and spatial analyses further indicate that immune exclusion in osteosarcoma is heterogeneous and dynamic rather than uniform or static (72, 73). Different tumors, and even distinct regions within the same lesion, can exhibit varying degrees of immune accessibility (74). This heterogeneity has direct implications for therapeutic response, supporting the view that immune exclusion lies along a continuum and may be partially reversible under appropriate conditions. Recognizing immune exclusion as a state-level phenomenon is therefore important for avoiding overly deterministic interpretations of immune resistance (75).

Despite recent progress, several limitations remain. Most available studies rely on cross-sectional profiling, providing limited insight into how immune exclusion evolves under therapeutic pressure (76). Longitudinal and spatially resolved analyses will be required to clarify whether exclusion precedes resistance or is reinforced during treatment (8). In addition, although multi-omics signatures associated with immune exclusion are increasingly reported, their causal contributions to maintaining immune inaccessibility still require further functional validation.

From a translational perspective, immune exclusion represents both a barrier and an opportunity (77). Durable re-sensitization in osteosarcoma is unlikely to arise from intensified cytotoxic therapy alone, and may instead depend on restoring immune access and engagement. In this framework, immune exclusion functions as a gatekeeper state that determines whether downstream immune-based or combination strategies can succeed. Targeting immune exclusion therefore offers a rational immunological framework for addressing treatment resistance in osteosarcoma.
